# Supplementary material for: The Role of Needle Fear in Pediatric Flu Vaccine Hesitancy: A Cross-Sectional Study in Bologna Metropolitan Area
Source: Vaccines (Basel). 2022 Aug 25;10(9):1388. doi: 10.3390/vaccines10091388 (PMC9506010; doi:10.3390/vaccines10091388)
Supplement: Supplementary file 1 [file vaccines-10-01388-s001.zip › vaccines-1874711-supplementary.pdf]

Supplementary Material

Questionnaire (Translated from Italian)

Q.1: “Are you a parent/guardian? Y or N”
Q.2: “Do you live in the Bologna Metropolitan Area? Y or N”
Q.3: “What is your gender? M, F or Other”
Q.4: “How old are you?”
Q.5: “What is your level of education? Middle School or lower; High School; University degree”
Q.6:” When was your son/daughter born?”
Q.7:” Do you have any 60+ year old family members? Y or N”
Q.8: “How often does your son/daughter spend time with those family members? Rarely (once a week or less); Sometimes (more than once a week, but less than one hour a day); Often (at least one hour a day); Never”
Q.9: “Do you have any family members with chronic diseases (e.g.diabetes, cardiovascular, pulmonary, neurological diseases, cancer etc.)? Y or N”
Q.10: “How often does your son/daughter spend time with those family members? Rarely (once a week or less); Sometimes (more than once a week, but less than one hour a day); Often (at least one hour a day); Never”
Q.11: “Did your son/daughter get fully vaccinated with all mandatory/recommended vaccinations? Y or N”
Q.12: “Did your son/daughter get vaccinated during the 2021-2022 flu campaign? Y or N”
Q.13: “Is your son/daughter affected by any chronic diseases? (e.g. neurological, pulmonary, cardiovascular, cancer, diabetes, etc.)? Y or N”
Q.14: “Has your child ever shown fear/discomfort caused by the needle during a vaccination? Y or N”
Q.15: “Has your pediatrician ever talked about/proposed/recommended flu vaccination for your child? Y or N”
Q.16: “Have you ever heard of the possibility of pediatric flu vaccination in your community? Y or N”
Q.17: “Do you think you will get your son/daughter vaccinated against influenza in the future? Y or N”
Q.18: “Would you prefer to have your son/daughter vaccinated if the route of administration was a nasal spray instead of injection? Y or N”

Table S1 – Association between covariates and “Would consider non-injective vaccination” variable

	OR	95% C.I.	*p*-value
**Fear 1**	3.79	1.63 - 9.43	0.003
**Awareness 1**	0.51	0.21 - 1.19	0.013
**Pediatrician advice 1**	0.60	0.22 - 1.51	0.028
**Parents gender 1**	0.81	0.33 - 1.99	0.645
**Parents age**	0.99	0.94 - 1.05	0.803
**Parents edu 1**	0.81	0.30 - 2.19	0.685
**Parents edu 2**	0.85	0.32 - 2.26	0.744

Analysis performed on subgroup of parents/guardians that responded that they would not get their children vaccinated in the future
